# Τ cell-mediated adaptive immunity in the transition from metabolic dysfunction-associated steatohepatitis to hepatocellular carcinoma

**DOI:** 10.3389/fcell.2024.1343806

**Published:** 2024-05-07

**Authors:** Grigorios Papadopoulos, Eirini Giannousi, Aikaterini P. Avdi, Rallia-Iliana Velliou, Polyxeni Nikolakopoulou, Antonios Chatzigeorgiou

**Affiliations:** ^1^ Department of Physiology, Medical School, National and Kapodistrian University of Athens, Athens, Greece; ^2^ Department of Neuroscience, Karolinska Institute, Stockholm, Sweden; ^3^ Center for the Advancement of Integrated Medical and Engineering Sciences (AIMES), Karolinska Institute and KTH Royal Institute of Technology, Stockholm, Sweden

**Keywords:** adaptive immunity, T cells, metabolic dysfunction-associated steatotic liver disease (MASLD), metabolic dysfunction-associated steatohepatitis (MASH), hepatocellular carcinoma (HCC), *in vivo* models, immunotherapies

## Abstract

Metabolic dysfunction-associated steatohepatitis (MASH) is the progressed version of metabolic dysfunction-associated steatotic liver disease (MASLD) characterized by inflammation and fibrosis, but also a pathophysiological “hub” that favors the emergence of liver malignancies. Current research efforts aim to identify risk factors, discover disease biomarkers, and aid patient stratification in the context of MASH-induced hepatocellular carcinoma (HCC), the most prevalent cancer among MASLD patients. To investigate the tumorigenic transition in MASH-induced HCC, researchers predominantly exploit preclinical animal-based MASH models and studies based on archived human biopsies and clinical trials. Recapitulating the immune response during tumor development and progression is vital to obtain mechanistic insights into MASH-induced HCC. Notably, the advanced complexity behind MASLD and MASH pathogenesis shifted the research focus towards innate immunity, a fundamental element of the hepatic immune niche that is usually altered robustly in the course of liver disease. During the last few years, however, there has been an increasing interest for deciphering the role of adaptive immunity in MASH-induced HCC, particularly regarding the functions of the various T cell populations. To effectively understand the specific role of T cells in MASH-induced HCC development, scientists should urgently fill the current knowledge gaps in this field. Pinpointing the metabolic signature, sketching the immune landscape, and characterizing the cellular interactions and dynamics of the specific T cells within the MASH-HCC liver are essential to unravel the mechanisms that adaptive immunity exploits to enable the emergence and progression of this cancer. To this end, our review aims to summarize the current state of research regarding the T cell functions linked to MASH-induced HCC.

## 1 Introduction

### 1.1 MASLD/MASH pathophysiology and the role of immunity

Metabolic dysfunction-associated steatotic liver disease (MASLD) affects almost one-third of the global population, posing an increased risk for the emergence of metabolic dysfunction-associated steatohepatitis (MASH) and MASH-induced hepatocellular carcinoma (HCC) (Younossi et al., 2023). MASH-induced HCC is a type of cancer tightly associated with the extent of fibrosis and the existence of cirrhosis in the liver, but it can also arise in the font of less progressed forms of MASLD (Ramachandran et al., 2019; Chrysavgis et al., 2022). While MASLD is primarily associated with metabolic dysfunction, the progression to advanced disease stages, including steatohepatitis and liver cancer, is significantly influenced by the immune processes within the liver (Peiseler et al., 2022). Although the innate immune responses retain a crucial role throughout MASH pathophysiology, increasing evidence pinpoints adaptive immunity as a major contributor to HCC development (Sutti and Albano, 2020; Hirsova et al., 2021; Ramadori et al., 2022; Petagine et al., 2023).

During the MASLD-to-MASH transition, the liver’s innate immune compartment is triggered via *de novo* lipogenesis-driven lipotoxic steatosis (Papadopoulos et al., 2023) and aberrant peroxidation of fatty metabolic intermediates (Seki et al., 2002). Concurrently, the liver microenvironment is changing dramatically; oxidative damage (Feldstein et al., 2003; Zhang et al., 2022b) and the number of senescent hepatocytes increase gradually (Papatheodoridi et al., 2020), while inflammatory cues (i.e., damage-associated molecular patterns, DAMPs) alter the liver’s microenvironment to foster disease progression and tissue damage. Therefore, a pro-inflammatory niche associated with the MASH phenotype is gradually shaped; increased activation of liver-infiltrated immune cells, mainly macrophages, contribute to augmented levels of several cytokines like TNFa, IL1-β, CCL2 and CCL5 (Sawada et al., 2023) and favor lobular inflammation (Takahashi, 2014), while hepatocyte ballooning underlines dysfunction in major lipolytic processes (Powell et al., 2021), often in the set of insulin resistance (Chatzigeorgiou and Chavakis, 2015). In the long term, deposition of extracellular matrix components is potentiated (Powell et al., 2021). These mechanisms highlight the importance of hepatic immune dynamics during the transient stages of MASLD and MASH and are the backbone of MASH pathophysiology preceding the MASH-induced HCC transition (Peiseler and Tacke, 2021; Huby and Gautier, 2022). However, the specific immunomodulatory characteristics directly linked to the transition of MASH into a tumorigenic state and the components of the hepatic immune system playing a decisive role before and during the transformation into HCC are currently unclear (Pinter et al., 2023).

Naturally, the hepatic innate compartment plays a significant role in maintaining immune homeostasis, a feature disturbed during liver cancer. For example, Kupffer cells (KC), the most frequent immune population in the liver, regulate immune tolerance through complex interactions with stromal and endothelial cells within the different zones of hepatic lobules (Crispe, 2014). Specific KC subtypes though, such as the M2-polarized tumor-associated macrophages (TAMs), are even more related to tumor initiation and development. Specifically, through secretion of growth factors such as vascular endothelial growth factor A (VEGFA) and via deposition of extracellular matrix (ECM) components such as matrix metalloproteinase 9 (MMP9), TAMs can contribute to neovascularization during HCC (Zhang et al., 2023), but might also enable immunosuppression (e.g., increased Tregs recruitment, inhibition of CD8+ T cell cytotoxicity) through C-C motif ligand (CCL)20 signaling (Wu et al., 2019). Additionally, other innate cell types like neutrophils, which can consist a source of pro-tumorigenic hepatic damage through NETosis (Van Der Windt et al., 2018), the dendritic cells (DC) whose periportal accumulation dictates priming of naïve cells (Deczkowska et al., 2021), and also the platelets, which can interact with liver KCs to enable recruitment of cytotoxic cells (Malehmir et al., 2019), might be participating in the tumorigenic transition of MASH. However, emerging evidence increasingly identifies T cells, along with their innate lymphoid counterparts and unconventional subtypes (e.g., MAIT, iNKT, γδ T cells), as the primary cell types underlying MASH-induced HCC (Zheng et al., 2021; Peiseler et al., 2022).

### 1.2 Liver remodeling during HCC development

Before discussing how T cells behave in the MASH-induced HCC tumor microenvironment (TME), it is important to underline certain features of liver remodeling exhibited during HCC. Initially, remodeling of the steatotic liver relies on inflammation in the course of early MASH, where macrovesicular steatosis gradually switches into lobular inflammation, followed by scar tissue formation towards varying grades of hepatic fibrosis (Powell et al., 2021). However, the transition from MASH to HCC implicates more complex changes in further functional levels apart of immunity (Anstee et al., 2019; Chrysavgis et al., 2022).

Specifically, imbalances in core signaling and developmental pathways, such as the fibrogenic Notch pathway, which acts in synergy with the Wnt/β-catenin axis, have been shown to orchestrate dynamic changes of hepatocellular fate, thus diversifying the variety of cell populations in the liver stroma during diet- and carcinogen-induced HCCs in mice (Zhu et al., 2021b); the respective studies have even used pathway-specific signatures to characterize distinct subtypes of MASH-associated tumors (Zhu et al., 2021a). Depending on its origin, the HCC tumor can provoke different forms of functional and structural remodeling of the extracellular matrix within the hepatic parenchyma (Ismail et al., 2020; Van Tienderen et al., 2023). For instance, stromal cell type redistribution appears typically in HCC, with trans-differentiation phenomena like epithelial-to-mesenchymal transition and endothelial-to-mesenchymal transition retaining an undefined, yet crucial role (Giannelli et al., 2016; Velliou et al., 2023).

Moreover, the liver tissue experiences significant alterations in typical intercellular relationships, such as the interactions of hepatic stellate cells (HSCs) with macrophages and myeloid-derived suppressor cells (MDSCs). Activated HSCs influence macrophages’ polarization fate or the expansion rate of MDSCs and potentially add immunosuppressive features to the TME during human and mouse HCC (Ji et al., 2015; Hsieh et al., 2019; Matsuda and Seki, 2020). Additionally, novel interactions can emerge, such as those between cancer-associated fibroblasts (CAFs) and hepatic immune populations (Ying et al., 2023). While the stem cell milieu contributes to increased proliferative capacity and stemness in the HCC liver (Zhu et al., 2021b; Yu et al., 2023), current research highlights a strong link between the MASH-induced HCC phenotype and the biological fonts of immune tolerance, suppression, and surveillance (Peiseler and Tacke, 2021; Riaz et al., 2022; Pinter et al., 2023). To date, experimental animal models are the gold standard in the MASH-induced HCC research field (Febbraio et al., 2019; Gallage et al., 2022). Recent *in vivo* studies highlight the significance of adaptive immunity, particularly T cells, recognizing their pivotal role in MASH-induced HCC tumor development (Hirsova et al., 2021; Ramadori et al., 2022). This review provides an overview of T cell functions in MASH to HCC transition, evaluates *in vivo* models, and briefly discusses the status of human MASLD/MASH ongoing immunotherapies.

## 2 T cells and the MASH- HCC transition

### 2.1 T cells sub-populations in MASH and MASH-induced HCC biological phenomena

In the context of liver disease, the biological features of immune tolerance, suppression and surveillance which were previously mentioned, deviate from MASLD’s and MASH’s innate immunity-centered phenotype. Instead, they are closely linked to the functions of various T cell populations within the cancerous liver (Sharma et al., 2015; Pfister et al., 2021; Tang et al., 2022). Therefore, T cells are now recognized as a predominant immune population in the MASH-induced HCC transition and pathogenesis (Ramadori et al., 2022).

T cells perform various physiological roles in the liver even before the emergence of MASH. These functions include the immune surveillance of the hepatic tissue, aimed at detecting and eliminating exogenous antigens or infected hepatocytes (Lopez-Scarim et al., 2023), or even the retention of antigen-specific T cell tolerance, as it can occur upon the antigen cross-presentation mediated by liver sinusoidal endothelial cells (LSECs) (Limmer et al., 2000). Additionally, the secretory phenotype of different T cell populations in the liver including both T helper and T cytotoxic cell subtypes, can have a significant impact in maintaining hepatic metabolic homeostasis, such as normal levels of insulin sensitivity (Sim et al., 2023). Their pivotal role in more advanced stages of the disease, characterized by robust activation of adaptive immunity, often diminishes the recognition of their homeostatic function, which is overshadowed by innate immunity during early MASH. Nevertheless, as hepatic injury reaches certain thresholds in the MASH liver, the importance of T cells in disease progression escalates exponentially. Finally, the determinant of pathological damage lies in whether CD8+ T cells will promote resolution of fibrosis by retaining sufficient activation levels, but without aberrant non-specific induction of apoptosis towards hepatocytes (Hirsova et al., 2021). It is at this juncture that the T cell behavior and phenotype during MASH, might be classified as anti- or pro-tumorigenic (Hirsova et al., 2021; Li et al., 2022).

The main feature of MASH-induced carcinogenesis is that of T cell exhaustion, characterized by reduced capacity of T cells to activate immune responses and eliminate infected or cancerous cells (Blank et al., 2019). Although exhaustion of various T cell populations, including CD4+ and CD8+ T cells, has been previously observed in liver cancer (Zheng et al., 2017), the accumulation of exhausted CD8+ T cells stands out as a primary adaptive immune characteristic contributing to immunologic dysfunction during HCC (Dudek et al., 2021; Pfister et al., 2021). Interestingly, as it will be discussed next, *in vivo* models in the context of MASH-induced HCC and pre-cancerous MASH stages, highlight a population of exhausted CD8+ T cells which tend to adopt a CXCR6+ PD1^high^ phenotype, indicative of their tendency to act auto-aggressively (Dudek et al., 2021) and impair the liver’s immune surveillance (Pfister et al., 2021), both in humans and mice. Nevertheless, CXCR6+ CD8+ T cells are responsible for crucial perturbations of the liver even from the early MASH stages, where their number and residency are regulated by IL-15 signaling (Dudek et al., 2021). This exhausted T cell subset is activated as a response to several immune (e.g., tumor necrosis factor, TNF) or metabolic stimuli (e.g., acetate) and can induce hepatocyte apoptosis through FAS/FASL (Yahoo et al., 2023). Therefore, a significant increase in the number of hepatic CXCR6+ CD8+ T cells might exaggerate damage in the liver parenchyma, aggravate MASH and ultimately, imbalance immunity and weaken liver’s defenses against cancer. In general, MASH seems to interfere with the anti-tumoral coordination of T cells; studies suggest that CD4+ and CD8+ T cells are impacted in different ways during MASH. For instance, CD4+ T cells may become prone to depletion (Heinrich et al., 2021), while CD8+ T cells may lose their mitochondrial wellness (Wabitsch et al., 2022) and as of that, they can impede the effectiveness of anti-cancer treatments that depend on them (e.g., anti-PD-1 therapies that are based on CD8+ T cell responses).

The increased levels of exhausted T cells along with the reduced immunosurveillance observed in MASH-induced HCC, might lead to insufficient resources for shaping strong anti-tumor immune responses in the liver. As showed by Inada et al. (2019), tumor-associated antigen (TAA) -specific T cell responses were less abundant in human MASH-induced HCC compared to other etiology HCCs. Furthermore, the study noted that an increased number of CTLA-4 -expressing CD8+ T cells was inversely correlated with the presence of TAA responses in the system of MASH-HCC patients (Inada et al., 2019). Accordingly, targeting the CTLA-4+ CD8+ T cell population with anti-CTLA-4 antibodies in immunoassay screenings, successfully restored the intensity of TAA responses in samples from MASH-HCC patients (Inada et al., 2019). These findings suggest that MASH-induced HCC, along with other HCC subtypes, could involve differential abundance in specific T cell subpopulations, which could in turn be essential in identifying therapeutic targets based on the distinct HCC subtype.

On the other hand, T-regulatory cells (Tregs) might have opposing contributions to MASH-induced HCC. While their presence is deemed beneficial in humans with viral HCC (Sharma et al., 2015), murine studies suggest that an imbalanced metabolic phenotype of Tregs can exacerbate the loss of immunosurveillance, promoting the tumorigenic shift of MASH (Wang et al., 2021; Riaz et al., 2022). In addition, distinct CD4+ T cell subsets, such as Th1 and Th17 helper T cells, which contribute to MASLD to MASH progression (Her et al., 2020), might have a critical role in the MASH to HCC transition. Of note, animal studies suggest that metabolic changes occurring during, but also before the emergence of MASH-induced HCC, such as the increased fatty-acid oxidation that compensates for aberrant steatosis, may lead to progressive mitochondrial-ROS -induced apoptosis of CD4+ T cells, hindering antitumor immunity (Ma et al., 2016; Brown et al., 2018). Further exploration of subsets like Th1, Th2, and Th17 helper T cells in MASH-induced HCC necessitates studying their immune dynamics and alterations during the transition.

Finally, innate T cells, such as invariant natural killer T cells (iNKT) and mucosal-associated innate T cells (MAIT), play a pivotal role in the pathogenesis of MASH and MASH-induced HCC. These cells are known to reside to a greater extent in the liver compared to other tissues, and have an indispensable role in several immunological, as well as metabolic processes that are decisive during different stages of the MASLD spectrum. Both iNKTs and MAIT cells can exploit environmental factors (such as dietary metabolites) or endogenous metabolites of the liver through their T cell receptor (TCR), and shape responses that might have a beneficial or detrimental impact for the liver (Papanastasatou and Verykokakis, 2023). Evidence has shown that these cells can exhibit a pro-tumorigenic role, attributed to excessive production of certain cytokines associated with tumor progression (e.g., IL-8 in the case of MAIT) (Duan et al., 2019) or increased fibrogenesis (e.g., potentiated by iNKT-secreted CXCR6) (Wehr et al., 2013). On the other hand, their impaired function or their insufficient amount, has also been correlated with the emergence and progression of HCC (Papanastasatou and Verykokakis, 2023), making them a “double-edged sword” for liver health; for instance, two opposing findings not to come as unexpected are that lipid-induced senescence of iNKTs has a deleterious impact during certain cases of HCC (Cheng et al., 2023), however, increased numbers of iNKTs have been positively correlated with the severity of steatosis during MASLD (Adler, 2011).

### 2.2 Preclinical *in vivo* MASH-induced HCC models and T cell-mediated adaptive immunity

**FIGURE 1:**
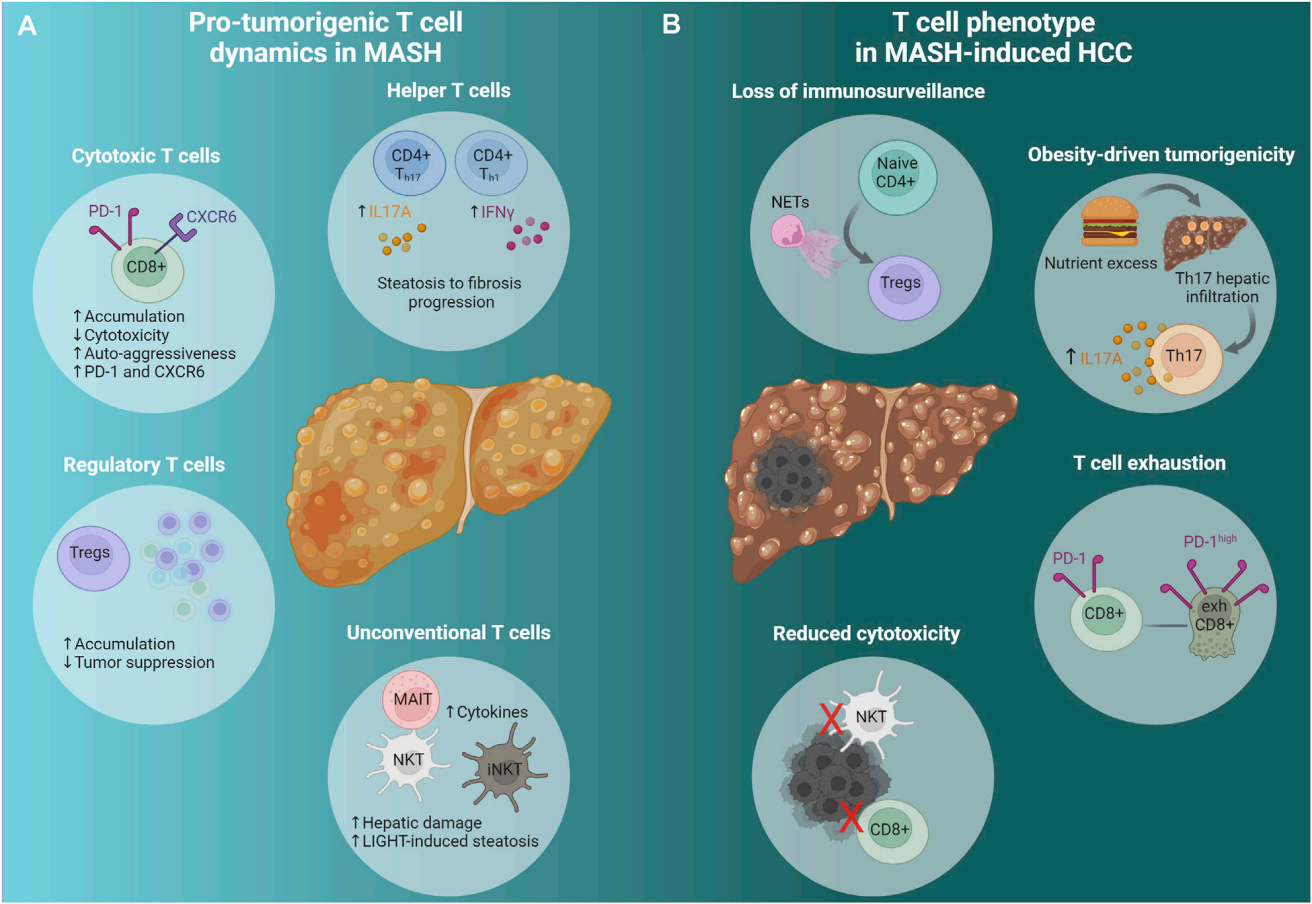
Adaptive immunity in advanced MASH and MASH-induced HCC. **(A)** Pro-tumorigenic T cell dynamics in MASH. A cascade of events predisposes the liver microenvironment to foster tumor formation and development. Conventional (CD8+, CD4+) and unconventional (NKT, iNKT, MAIT) T cells change dynamically to promote HCC emergence; T_h_ cells promote fibrosis progression, cytotoxic T cells (CXCR6+ CD8+ PD-1^high^) display diminished activity and strong auto-aggression, while they express high levels of PD-1 and CXCR6. Tregs accumulate leading to reduced immunosurveillance. Unconventional T cells, namely MAIT and iNKT, produce pro-tumorigenic cytokines that promote tumorigenesis and hepatic damage respectively. NKT cells initiate MASH and HCC development via the excessive secretion of LIGHT, a LTβR ligand. **(B)** T cell phenotype in MASH-induced HCC. NETs promote naive CD4+ differentiation to Tregs and facilitate Treg activation resulting in immunosurveillance suppression. Hepatocarcinogenesis due to nutrient excess is attributed to Th17 cells’ hepatic infiltration and excessive IL-17A production. The accumulation of exhausted activated CXCR6+ CD8+ PD-1^high^ cells contributes to HCC, while NKT and CD8+ T cells have impaired cytotoxic function. See also Section 2.2 and Table 1. Created via Biorender.com. MASH, metabolic dysfunction-associated steatohepatitis; HCC, hepatocellular carcinoma; CD4+ T_h17_, CD4+ T helper 17 cells; CD4+ T_h1_, CD4+ T helper one cells; IL17A, Interleukin 17A; IFNγ, interferon gamma; CD8+, cytotoxic T cells; PD-1, programmed cell-death protein 1; CXCR6, C-X-C chemokine receptor type 6; Tregs, regulatory T cells; MAIT, mucosal associated invariant T cells; NKT, natural killer T cells; iNKTs, invariant natural killer T cells; LIGHT, tumor necrosis factor superfamily member 14; NETs, neutrophil extracellular traps; Naive CD4+, naive CD4+ T cells; exhCD8+, exhausted CD8+ T cells.

Animal models represent one of the most realistic tools for simulating antitumor immune responses and studying the ME characteristics in MASH-induced HCC. Herein, we aim to summarize studies that utilized *in vivo* models to mimic MASH-induced HCC and explore the role of adaptive immunity cell populations during the initiation of MASH-induced HCC (see Table 1; Figure 1). Researchers employ diverse murine models intending to reproduce key human disease traits regarding inflammatory events, histopathological alterations, metabolic reprogramming, hepatocyte function, and hepatic immune microenvironment. These models are generated through diverse dietary interventions, chemical treatments, genetic modifications, and transplantation of HCC cells in the liver. Dietary models, while having a low tumor development rate, recapitulate the histological, physiological, and metabolic features of human MASH and MASH-induced HCC (Anstee et al., 2019; Farrell et al., 2019; Gallage et al., 2022). Chemical-induced models and HCC cell orthotopic models are usually combined with a specific diet, resulting in rapid and advanced carcinogenesis in the liver. However, some models lack vital traits of the MASH phenotype, namely, obesity and glucose intolerance (Anstee et al., 2019; Farrell et al., 2019; Gallage et al., 2022). Also, genetically modified models combined with a modified diet mimic MASH development and transition to HCC by exhibiting a more severe disease phenotype (Anstee et al., 2019; Farrell et al., 2019; Gallage et al., 2022). Integrating these models with genetic modifications altering or diminishing specific T cell function helps characterize the protective and pro-carcinogenic role of T cells during MASH development and HCC (Vesely et al., 2011; Ringelhan et al., 2018).

**TABLE 1:** Summary of the preclinical MASH-induced HCC models and the respective T cell functions.

Model	Sex	Genetic modifications	Intervention diet	Duration	Metabolic changes	HCC	T cells function	References
CD-HFD	M&F		CD-HFD; 44.9% fat (4.74 kcal/g), 20% proteins, 35.1% carbohydrates	12 months	↑ Body weight	Yes	MASH and HCC pathogenesis is regulated by hepatocellular LtβR and canonical NF-κB signaling pathways, that modulate the interplay between CD8+ T cells, NKT cells and their cytokines	Wolf et al. (2014)
					↑ Liver fat mass			
					↑ Triglycerides			
					↑ ALT, ↑ Cholesterol, hepatocyte ballooning, mallory denk bodies, steatosis, fibrosis, obesity, chronic metabolic syndrome			
	M		CD-HFD; 44.9% fat (4.74 kcal/g), 20% proteins	13 months	↑ Body weight, ↑ ALT	Yes	MASH-related hepatic damage due to accumulation of exhausted activated CD8+PD1+ T cells impairs immune surveillance in the HCC-liver during immunotherapy	Pfister et al. (2021)
			35.1% carbohydrates		↑ NAS score, steatosis, fibrosis			
	M	METTL3 Knock in/out	CD-HFD; 36.2% fat, 27.2% protein, 27.3% carbohydrate, without choline + DEN injection	27 weeks	↑ Cholesterol biosynthesis	Yes	METTL3-SCAP mediated cholesterol biosynthesis impairs CD8+ cytotoxicity in MASH-induced HCC	Pan et al. (2023)
					↑ Serum cholesterol			
					↑ Triglycerides, ↑AST, hyperlipidemia, liver damage			
HFD and CD-HFD	M		HFD; 18.4% protein, 23.5% fat (46% energy from fat), 4.7% fiber CD-HFD; without choline, 22.6% protein, 23.5% fat (43% energy from fat), 5.4% fiber	40 weeks	Hepatocyte ballooning, apoptotic-necrotic hepatocytes, steatosis, fibrosis, obesity, hepatic inflammation	Yes	TCPTP inactivation favors MASH through STAT-1 -induced T cell recruitment and promotes HCC via STAT-3 -dependent mechanisms, during obesity	Grohmann et al. (2018)
STAM	NI		STAM; HFD; 60% fat, 20% protein, 20% carbohydrate + Streptozotocin injection	20 weeks	Steatosis	Yes	During MASH to HCC transition NETs induce Treg activity and naïve CD4+ T cells differentiation to Tregs. Elevated Tregs lead to immunosurveillance suppression	Wang et al. (2021)
			CD-HFD; 58.0% fat, 16.4% protein, 25.5% carbohydrate, without choline + DEN injection	16 weeks	Steatosis	Yes		
hURI-tetOFF^hep^	NI	Hepatic hURI Knock in	HFD; 45% fat, 20% proteins, 35% carbohydrates	65 weeks	Inflammation, hepatic steatosis with Mallory-Denk bodies, steatosis, fibrosis, and liver injury	Yes	Hepatic overloading with nutrients potentiates tumorigenicity during MASLD/MASH in a URI-dependent manner, relying on Th17-mediated inflammation and IL17A signaling as a response to genotoxic stress	Gomes et al. (2016)
MYC-ON	NI	MYC oncogene	MCD; without methionine and choline, 40% high sucrose, 10% fat + with 10% w/w corn oil	14 weeks	Hepatic lipid accumulation	Yes	Obesity-induced lipid and ROS accumulation leads to intrahepatic CD4+ T cells reduction and hepatocarcinogenesis	Ma et al. (2016)
MUP-uPA	M	Hepatocyte uPA overexpession	HFD; 59% fat, 15% protein, 26% carbohydrates	11 months	Steatosis, fibrosis, obesity, ↑ ALT	Yes	Accumulation of liver-resident IgA + cells during MASH suppress liver cytotoxic CD8+ T cells and promote HCC	Shalapour et al. (2017)
			STAM; HFD; 60% fat, 20% protein, 20% carbohydrate + Streptozotocin injection	25 weeks	Steatosis, lipid accumulation, ↑ ALT	Yes		
MCD + Hep55.1c-Tet3G-LucOS HCC cells	F		MCD;without methionine and choline, 40% high sucrose, 10% fat + 2 mg/mL of doxycycline + 10% glucose of drinking water	30 days	NI	Yes	Accumulation of liver macrophages impaired antitumor CD8+ T cell activity/immunity	McVey et al. (2022)
	NI	MYC oncogene	MCD; without methionine and choline, 40% high sucrose, 10% fat	11 weeks	NI	Yes	Inhibition of CPT rescued intrahepatic CD4+ T cell apoptosis and prevented MASH-induced HCC	Brown et al. (2018)
HFD + RIL-175-LV-OVA-GFP HCC cells	NI		HFD; 45% kcal fat, 15% kcal protein, 41% kcal carbohydrate	30 weeks	↑ Body weight, ↑ NAS score, steatosis	Yes	High levels of CD44+ CXCR6+ PD-1^high^ CD8+ T cells lower immune activity and tumor surveillance	Lacotte et al. (2023)
HFHC + RIL-175 HCC cells	M		HFHC; 58 kcal% fat and Sucrose + drinking water with HFCS	17 weeks	Obesity, steatosis, fibrosis, ↑ triglycerides, ↑ hepatic cholesterol accumulation	Yes	Excessive cholesterol metabolic signaling reduces tumor-infiltrated NKT cells and prevents NKT cell-driven antitumor immunosurveillance	Tang et al. (2022)

HCC, column: Yes/No indicates if tumors were detected in the liver.

(NI, no information given; M, male; F, female; CD-HFD, choline deficient high-fat diet; ALT, alanine transaminase; NAS, NAFLD activity score; HFD, high-fat diet; TCPTP, T cell protein tyrosine phosphatase; STAM, stelic animal model; DEN, diethylnitrosamine; NETs; neutrophil extracellular traps; AST, aspartate aminotransferase; SCAP, cleavage-activating protein; hURI, human hepatic unconventional prefoldin RPB5 interactor (URI); MYC-ON, liver-specific MYC oncogene transgenic mice; MCD, methionine choline deficient; ROS, reactive oxygen species; uPA, urokinase plasminogen activator; IgA+, immunoglobulin-A-producing cells; CPT, carnitine palmitoyl transferase; HFHC, high-fat high-carbohydrate; HFCS, high fructose corn syrup.

#### 2.2.1 Dietary murine models

A widely utilized dietary scheme to investigate MASH and MASH-induced HCC pathogenesis is that of choline-deficient diet supplemented with high levels of fat, known as the choline-deficient high-fat diet (CD-HFD). Wolf et al. (2014) revealed the interaction between the CD8+ T cells, natural killer T cells (NKT), their secreted cytokines, and hepatocytes during MASH and HCC development. Specifically, in mice fed a CD-HFD diet for 12 months hepatocytes, via a cooperative manner of the LTβR and the canonical NF-κB signaling pathway, activate CD8+ T cells and NKT cells, which further cause liver damage, steatosis, MASH development, and ultimately transition to HCC. Antibody mediated deletion of CD8+ T cells ameliorated liver damage but did not influence lipid metabolism, since serum cholesterol values did not alter. On the other hand, NKT cells via the elevated LIGHT secretion mediate fatty acids uptake, activate HSCs, and enhance liver damage. Both CD8+ and NKT cells display increased secretion of proinflammatory cytokines, i.e., TNF superfamily cytokines, TGF-β, IFN-γ, and IL1-β, that promoted liver damage and transition to HCC. Therefore, Wolf et al. (2014) proposed targeting specific signaling pathways as possible therapeutic alternatives since they trigger MASH and HCC development.

Moreover, Pfister et al. identified an increased number of exhausted, activated CD8+PD1+ T cells in mice fed a CD-HFD; deletion of these cells in mice suffering from MASH prevented HCC incidence and provided additional evidence for the pro-tumorigenic role of CD8+ T cells (Pfister et al., 2021). Specifically, mice on a 13-month-long CD-HFD diet displayed an accumulation of exhausted unconventionally activated CD8+PD1+ T cells that favor the MASH to HCC transition by inducing severe hepatic injury and impaired anti-PD1 immunotherapy response. In parallel, therapeutic PD1-or PDL1-related immunotherapy increased CD8+PD1+ T cells within tumors and failed to regress tumor progression. These findings suggest that the liver microenvironment during the MASH to HCC transition affects the efficacy of tumor surveillance and immunotherapy response (Pfister et al., 2021). Moreover, mice that were fed a high fat diet (HFD) or a CD-HFD and became obese displayed increased oxidation levels of several protein tyrosine phosphatases (PTPs) indicating a potential role of their oxidation and inactivation in MASLD and MASH development (Grohmann et al., 2018). In parallel, Grohmann et al. linked the suppression of JAK/STAT PTPs with increased STAT-1 and STAT-3 signaling. In detail in obese mice a T cell protein tyrosine phosphatase (TCPTP) ablation, enhanced STAT-1 and STAT-3 signaling leading to MASH and HCC formation. The reduction of the elevated STAT-1 signaling in hepatocytes via a transgenic mouse attenuated the recruitment of activated cytotoxic T cells, leading to amelioration of hepatic inflammation and fibrosis, without preventing tumor development. On the contrary the correction of STAT-3 enhanced signaling, repressed tumor incidence irrespective of T cell recruitment, MASH, and fibrosis (Grohmann et al., 2018). Interestingly, a growing body of evidence renders STAT-3 signaling a pivotal mechanism in developing various malignancies (Poser et al., 2019; Fu et al., 2023). Grohmann et al. highlights that the recruitment of T cells and the development of MASH and fibrosis are not mandatory for developing HCC in obesity. Nevertheless, the oxidative and inflammatory microenvironment that prevails in MASLD could initiate tumorigenesis (Grohmann et al., 2018). These findings provide insights into potential mechanisms contributing to the increasing prevalence of HCC in non-cirrhotic livers associated with MASLD.

Another study scrutinizes the role of Tregs and neutrophil extracellular traps (NETs) in developing MASH-induced HCC. Specifically, Wang et al. used several murine models of MASH-induced HCC, including a CD-HFD in combination with diethylnitrosamine (DEN) injection and the stelic animal model (STAM) (Wang et al., 2021). In these models, they report decreased levels of CD4+ T cells but an increased frequency of Tregs and NETs that shape an immunosuppressive and pro-tumorigenic microenvironment. The ablation of Tregs in a STAM fed transgene “depletion of regulatory T cell (DEREG)” murine model resulted in a significant reduction of steatosis, fibrosis, and tumor incidence. This study elucidates how NETs interact with naive CD4+ T cells through TLR4 on their membrane and promote naive CD4+ T cell differentiation into Tregs, hence suggesting a new mechanistic pathway as a potential therapeutic target (Wang et al., 2021).

Lastly, to elucidate the role of METTL3 in MASLD and MASH, Pan et al. (2023) used a CD-HFD-DEN murine model with genetic modification of METTL3 expression. Specifically, liver-specific METTL3 knockin led to increased levels of cholesterol, impaired CD8+ T cell cytotoxicity, and promoted MASH-induced HCC development. On the other hand, knockdown of METTL3 enhanced CD8+ T cytotoxicity, accompanied by elevated IFN-γ and granzyme production, which blocked hepatic tumorigenesis (Pan et al., 2023).

#### 2.2.2 Genetically modified and dietary murine models

Researchers developed murine models combining specific genetic modifications with dietary interventions to better resemble the human MASH-induced HCC. Specifically, in a mouse with hepatocyte specific expression of the human unconventional prefoldin RPB5 interactor (URI), namely, the URI-tetOFF^hep^ mouse model that mimics nutrient excess by URI overexpression, they identified a pro-tumorigenic role for Th17 cells (Gomes et al., 2016). Excessive hepatic DNA damage due to URI expression led to extensive production of intrahepatic IL-17 induced by the selective recruitment of Th17 cells and the induction of extensive metabolic inflammation, liver damage, and hepatocarcinogenesis. Blocking of IL-17A signaling using an antibody against IL-17A ameliorated liver injury and HCC development; hence, IL-17A may serve as a valuable non-invasive marker for insulin resistance and the pathogenesis of MASH in obese patients (Gomes et al., 2016).

Furthermore, a well-established mouse model mimics the development of MASH and progression to HCC by overexpressing the proto-oncogene c-Myc (MYC-ON) specifically in hepatocytes, combined with a methionine-and-choline deficient diet (MCD) diet. Utilizing this model, Ma et al. (2016) showed the alterations in the T cell population during MASLD to HCC transition. MYC-ON MCD mice with MASLD displayed fewer intrahepatic CD4+ T cells, while intrahepatic CD8+ T cells remained stable. Depleting CD4+ T cells increases hepatic tumor lesions in MYC-ON MCD mice and accelerates hepatocarcinogenesis, thus confirming their anti-tumoral role (Ma et al., 2016). The same results were observed in additional experiments with mice fed choline-deficient L-amino acid (CDAA) or HFD, both in tumor-free and tumor-bearing conditions. These findings indicate that the dysregulated lipid metabolism in the liver, a MASLD hallmark, affects CD4+ but not CD8+ T cells. They also demonstrate that the hepatic CD4+ T cells have higher mitochondrial mass than CD8+ T cells and yield higher levels of mitochondria-derived reactive oxygen species (ROS). *In vivo* blockade of ROS with N-acetylcysteine (NAC) treatment ameliorated the CD4+ T cell reduction and delayed the onset of MASH-induced HCC. This study unraveled a connection between diet-induced lipid accumulation and compromised antitumor surveillance (Ma et al., 2016).

Shalapour et al. revealed an altered role of CD8+ T cells in mediating immunosurveillance against MASH-induced HCC. They utilized two murine models very similar to human MASH-induced HCC: a) the HFD MUP-uPA mice, in which overexpression of urokinase plasminogen activator induces ER stress in hepatocytes (Nakagawa et al., 2014) and b) the STAM mice, in which a streptozotocin injection combined with HFD is applied (Fujii et al., 2013). Using this combinatorial approach, the authors observed an accumulation of liver-resident immunoglobulin-A-producing (IgA+) cells in the fibrotic liver, which express programmed death ligand 1 (PD-L1) and interleukin-10 and directly suppress liver cytotoxic CD8+ T cells, thus blocking their immunosurveillance function. Eliminating CD8+ T cells expedited the development of hepatocellular carcinoma. On the other hand, ablation of IgA + cells hindered liver carcinogenesis and triggered the regression of established tumors through cytotoxic T cells (Shalapour et al., 2017).

#### 2.2.3 Orthotopic murine models

Besides the aforementioned murine models that mimic MASH-induced HCC through dietary or genetic interventions, studies have utilized orthotopic murine models combined with different dietary plans (McVey et al., 2022). Specifically, McVey et al. created a novel doxycycline-inducible antigen presenting HCC cancer cell line that was seeded into mouse livers following the MCD diet. Using this model, they found that MASLD impaired antitumor CD8+ T cell immunity against HCC. Immune profiling confirmed the accumulation of macrophages in the liver microenvironment, while their depletion, using clodronate liposomes, reversed the CD8+ T cell diminished antitumor activity (McVey et al., 2022). Thus, the authors concluded that the accumulated macrophages in the TME caused CD8+ T cell dysfunction.

Brown et al. investigated intrahepatic CD4+ T cells during MASLD and HCC by focusing on the regulation of carnitine palmitoyl transferase (CPT) genes, which control the mitochondrial β-oxidation of fatty acids and act as critical molecules in lipid catabolism (Brown et al., 2018). CPT genes were upregulated in hepatic CD4+ T cells in MASLD, resulting in greater ROS and CD4+ T cell apoptosis. Using perhexiline, a CPT inhibitor, in MYC-ON MCD-fed mice that developed HCC, they rescued intrahepatic CD4+ T cells and prevented MASH-induced HCC development. Their study confirms that CPT genes may act as putative therapeutic targets, which, via restoring CD4+ T activity, could aid MASH-induced HCC immunotherapy (Brown et al., 2018).

Lacotte et al. (2023) utilized HFD mice to assess tumor-specific immunity in MASH mice. Mice were fed a HFD for 30 weeks, developed MASH and showed increased CD44+ CXCR6+ PD-1+ CD8+ T cells in the liver (Lacotte et al., 2023). Upon intrahepatic injection of RIL-175-LV-OVA-GFP cells, a HCC cell line (RIL-175) expressing a non-self antigen, the ovalbumin protein, the MASH mice showed increased expression of OVA-specific CD8+ cells, which failed to prevent tumor growth. Looking into the TME, the OVA-specific CD44+ CXCR6+ CD8+ cells expressed higher levels of PD-1, suggesting a lower immune response; thus, in these mice, the immune system failed to prevent tumor growth. Treatment with an antibody against CD122 reduced CXCR6+ CD8+ PD-1^high^ T cells (Dudek et al., 2021); Lacotte et al. (2023) in their study, confirmed that after deletion of the CXCR6+ CD8+ PD-1^high^ T cells, the anti-CD122 treatment restored the tumor-specific T cell phenotype and prevented tumor growth. Thus, CD8+ T cell activity restoration was vital to prevent HCC growth; nevertheless, additional mechanisms that have acted synergistically cannot be excluded.

In an orthotopic high-fat high-carbohydrate (HFHC) murine MASH-induced HCC model, mice displayed inverse correlation between elevated cholesterol levels and reduced number of NKT cells both in the liver and the blood (Tang et al., 2022). Concurrently, HFHC mice after 17 weeks of feeding showed increased hepatic tumor weight, which is accompanied by decreased tumor infiltration of NKT cells. Restoring cholesterol levels by statin, ameliorated the NKT cell population, improved liver steatosis, and prevented obesity-promoted HCC development. The mTORC1/SREBP2 signaling pathway mediated the cholesterol accumulation in hepatocytes and the NKT dysfunction; thus, targeting this pathway could be an alternative approach to prevent and treat MASH-induced HCC (Tang et al., 2022).

MASH preclinical research employs numerous *in vivo* models to recapitulate the human MASH pathogenesis and phenotype. Due to the continuously increasing prevalence of MASH-induced HCC, scientists are utilizing existing or introducing new models to mimic disease development, progression, and, ultimately, HCC growth. The multifactorial nature of this disease places a heavy burden on preclinical models, each driving the disease pathogenesis through distinct mechanisms; several factors, including metabolic dysfunction, oxidative stress, lipotoxicity, and hepatic immune microenvironment heterogeneity, contribute to liver inflammation and determine T cell functionality. As shown in Figure 1, T cells play a crucial part in the progression of MASH-induced HCC since complex immune cell-mediated inflammatory processes are involved in disease development.

To date, the role of the two most prominent T cell subgroups in HCC development–CD4+ and CD8+ cells–causes scientific debate (Ringelhan et al., 2018; Ramadori et al., 2022); this issue arises from the fact that these cells are active throughout the disease timespan, possibly orchestrating different tissue responses that are disease stage-dependent. Moreover, the contradictions in the obtained results regarding the role of specific T cell subtypes may arise from innate differences among the utilized animal models.

Bearing in mind the numerous murine models analyzed in the present review, it is notable that there are several limitations. Firstly, the murine models present distinct metabolic and immune perturbations. Hence, they do not precisely mimic the human MASH and MASH-induced HCC since they may induce the disease by overcoming several MASH hallmarks, namely, obesity, insulin resistance, and adipose tissue inflammation. Moreover, studies lack the representation of both sexes and the extensive research of dietary and genetic interventions in both female and male mice. Most of the studies utilize only male mice, possibly due to the unsuccessful induction of the disease and the inconsistent phenotype that female mice often display (Gallage et al., 2022) (see Table 1). Moreover, orthotopic mouse models face several limitations, including the implantation of a high number of cancer cells in contrast to what is observed during carcinogenesis and the investigation of a strong antigenic immune response initiated only after HCC cell transplantation (McVey et al., 2022; Lacotte et al., 2023). Lastly, researchers focus on the function of one or a few T cell subtypes per study and not the coordinated interplay between the various T cell subsets during MASH and HCC progression. A comprehensive overview of the T cell interactions along the disease timespan, such as spatial proteomic analysis, is currently lacking.

In a nutshell, developing novel MASH-induced HCC models that closely resemble the human condition and address specific T cell roles is critical. A first step could be comparing the immune landscape between MASH-induced HCC patients and murine models. Next, it is vital to investigate thoroughly the interactions among a) the different immune cell subpopulations and b) immune cells and HSCs. Such an analysis would contribute further to the development of accurate preclinical MASH-induced HCC models, enabling the in-depth investigation of the mechanisms underlying T cell functionalities during disease progression and guiding immune regulatory interventions in HCC patients.

### 2.3 T cell-mediated adaptive immunity in human MASH-induced HCC and the path to immunotherapies

The burgeoning prevalence of MASLD and MASH, combined with the high risk of MASH-induced HCC development raises the need to exemplify the pathogenetic mechanisms of the disease. In contradiction to the *in vivo* models that showcase a prominent role of T cells for MASH-induced HCC pathogenesis, human-based studies that interpret the T cells functions and cellular interactions during the disease progression are scarce. Currently, only a few studies utilize liver biopsies from MASH-induced HCC patients as an initial screening of T cells phenotype. Specifically, in patients with MASH-induced HCC a significant accumulation of liver resident IgA+ cells was detected, that suppress CD8+ T cells cytotoxicity and tumor prevention (Shalapour et al., 2017). Tang et al. (2022) revealed that NKT cells reduction in MASH-induced HCC patients is correlated with hypercholesterolemia and impaired antitumor immunosurveillance. A more extensive study of Inada et al. highlighted the differences of the immune response of TAA–specific T cells and immune cell profile in three distinct groups of HCC patients categorized based on etiology, namely, patients with HBV-related HCC, HCV-related HCC or MASH-induced HCC (Inada et al., 2019). MASH-induced HCC patients show poor immune response to TAA although their immune profile is characterized by higher frequencies of effector regulatory T cells (eTregs) and CD8+ T cells strongly expressing CTLA-4 (Inada et al., 2019). Hence, there is an inverse correlation between these specific immune cell populations and immune response that can be detrimental for the immunotherapy efficacy in MASH-induced HCC.

Recently, studies have resorted to high-throughput experimental approaches, namely, RNA sequencing and spatial transcriptomics to characterize the TME in MASH-induced HCC. Spatial analysis revealed a heterogenous TME in MASH-induced HCC, where immune cells, including CD4+ and CD8+ T cells, Tregs, MDSCs and TAMs, were more abundant in the adjacent non-tumorous tissue areas compared to the tumors. These immune cell populations are presented with diverse phenotype, functions and abundance across different regions. In MASH-induced HCC the interaction of CD4+ and CD8+ T cells with MDSCs and TAMs leads to T cell exhaustion and immune evasion (Li et al., 2024). Furthermore, an extensive profiling of the MASH-induced HCC TME by Murai et al. (2023) unveiled an enriched immune TME, but with abundant exhausted T cells, high PD-L1 expression, M2 macrophages and CAFs infiltration. Utilizing a novel noninvasive imaging method, they show that MASH-induced HCCs with the aforementioned TMA are more susceptible to combined immunotherapy using anti–PD-L1 and anti-VEGF antibodies (Murai et al., 2023).

Even though there are limited data from human samples on the exact impact of T cells in MASH-induced HCC development, the landscape of HCC therapy is undergoing rapid evolution. Advancements in molecular targeted therapies and immunotherapies have revolutionized treatment strategies for advanced HCC. Among these, immune checkpoint inhibitors (ICIs) have emerged as one of the most promising approaches to combat HCC, since they initiate and enhance immune response in HCC patients (Llovet et al., 2022; Harrison et al., 2023). In the following lines, we will briefly outline the primary mechanisms employed by immunotherapies and highlight the current unmet needs in MASH-induced HCC therapeutic research.

Over the last years, the development of novel molecular targeted agents, namely, tyrosine kinase inhibitors (TKIs), with the combined rise of immunotherapies have significantly transformed the field of therapeutic strategies and improved the outcomes for HCC. Immunotherapies aim to counteract tumor-induced immunosuppression, primarily utilizing immune checkpoint inhibitors (ICIs). These ICIs are the predominant mechanism in effective immunotherapies by inhibiting key immune-checkpoints, namely, cytotoxic T lymphocyte antigen 4 (CTLA-4), programmed cell death 1 (PD-1), lymphocyte activation gene-3 (LAG-3), and T cell Ig mucin-3 (TIM3). Concurrently, CD28, glucocorticoid-induced TNFR-related protein (GITR), and OX40 (CD134) promote T cell expansion, also representing primary immunotherapy targets for HCC (Llovet et al., 2022). Among the first ICIs in clinical research are agents targeting PD1 and CTLA-4, which are expressed by T cells and negatively regulate immune responses. Agents such as nivolumab and pembrolizumab, monoclonal antibodies that block PD-1 and ipilimumab, a CTLA-4 inhibitor, have demonstrated good responses in HCC patients (Zhang et al., 2022a). Moreover, anti-CTLA4 treatment activates CD4+ and CD8+ cells in HCC patients (Agdashian et al., 2019). Besides the well-characterized PD-1 and CTL-4 ICIs, clinical research is now advancing on inhibiting additional co-inhibitory checkpoints like LAG3 (Chocarro et al., 2022) or TIM3 (Acharya et al., 2020), along with exploring various methods to enhance immune system activation (Cai et al., 2023). Combination of a PD-L1 inhibitor with TIM-3, LAG-3, or CTLA-4 inhibitor led to an upregulated *in vitro* tumor-infiltrating lymphocyte (TIL) response of HCC-derived T cells (Zhou et al., 2017).

Since MASH-induced HCC is characterized by high heterogeneity and complex pathogenetic mechanisms pertinent to immune regulation, DNA damage and oxidative stress, research interest has shifted towards the therapeutic combination of ICIs and molecular targets. An exciting recent development in HCC treatment involved the combination of an ICI, atezolizumab, with an anti-angiogenic medication, bevacizumab (an anti-VEGFA antibody). This combination surpassed the effectiveness of sorafenib, an inhibitor of multiple kinases involved in tumor growth and angiogenesis and utilized as systemic therapy, by improving overall survival rates from ∼11–14 to 19 months for patients with advanced HCC (Finn et al., 2020). Moreover, ongoing trials are testing the efficacy of relatlimab, a LAG-3 inhibitor and activator of exhausted T cells, combined with nivolumab (commercially available as Opdivo), to treat immunotherapy naïve patients that have undergone first-line TKIs (e.g., sorafenib) (Bhatt and Wu, 2023). Lastly, an alternative path is that of vaccine and cell therapy development. The successful implementation of such approaches has been proven challenging due to the immunosuppressive microenvironment of HCC, while efforts to suppress regulatory T cell infiltration pharmacologically have been unsuccessful (Greten et al., 2010). Clinical data from trials using genetically engineered T cells for HCC is limited to phase I studies. Preliminary data suggest that TCR-engineered T cells targeting alpha-feroprotein (AFP) (Goyal et al., 2019) and chimeric antigen receptor T cells (CAR-T) cells targeting CD133 (Dai et al., 2020) or GPC3 (Shi et al., 2020) show some early signs of antitumor activity. Furthermore, another trial investigated the potential of iNKT (CD1d restricted T cells) to inhibit HCC growth by promoting antitumor immunity (Guo et al., 2023). Patients with unresectable HCC after transarterial chemoembolization (TACE) failure received infused iNKT cells combined with transarterial embolization (TAE) which protects iNKT cell function. Patients with the double therapeutic scheme of iNKT cells infusion and TAE presented improved progression-free survival than those who received TAE alone. The iNKT cells-TAE treatment showed acceptable toxicity levels and) improved quality of life (Guo et al., 2023).

Although immunotherapies for HCC are advancing rapidly, the aforementioned studies and clinical trials recruit patients with HCC of various etiology (Llovet et al., 2023). Patient cohorts are often categorized as viral (HBV, HCV) or non-viral HCC, the latter including individuals with alcohol related disease, ΜASH and other etiologies. As a result, there is a lack of conclusive evidence regarding the effectiveness of immunotherapies specifically in MASH-induced HCC patients. Furthermore, to date no immunotherapies have been deployed for MASH that could be used as a hint for a novel MASH-induced HCC therapy (Anstee et al., 2019; Llovet et al., 2023). Even though the introduction of more MASH-induced HCC patients in clinical trials requires stricter inclusion criteria and may affect the patient recruitment, it is crucial to better stratify patients based on etiology and to consider specifically MASH-induced HCC as a standalone entity in the future clinical trials. Additionally, the exhausted and dysfunctional immune microenvironment in ΜASH-induced HCC as has been indicated by the numerous *in vivo* models (Table 1) and patients’ studies give rise to the question about the efficacy of immunotherapies. Two studies suggest that immunotherapies are more effective in viral-related HCC that in MASH-induced HCC (Haber et al., 2021; Pfister et al., 2021).

The development of animal models aiming at accurately replicating the intricate interplay between adaptive immunity and liver cells during carcinogenesis is a crucial step in designing novel and specific therapies for MASH-induced HCC. The *in vivo* models utilized (Table 1) offer unique insights into the pathogenetic mechanisms and cellular interactions of adaptive immunity during MASH to HCC transition. Nonetheless, they do not fully simulate the integrated and complex pathogenesis, microenvironment and heterogeneity of MASH-induced HCC. Future directions should aim towards the establishment of an immunocompetent humanized animal model that combines the basic traits of MASH that lead to HCC with the dynamic alterations of the immune microenvironment that occur during the disease progression. While numerous trials are in their initial stages, inspired by preclinical animal models, it is crucial for researchers to thoroughly examine past setbacks to enhance the translatability of the models and streamline the refinement of clinical trials. Such an approach, in turn, will pave the way for more effective, patient-oriented therapeutics through translational research.

## 3 Discussion

MASH-induced HCC is stepwise recognized as a distinct cancerous condition. To date, we have managed to dissect the role of several immune cell types on liver physiology during MASLD and MASH and assess their possible implications for the MASH tumorigenic transition (HCC) (Huby and Gautier, 2022). The contribution of adaptive immunity in MASH-induced HCC pathogenesis is currently acknowledged as a core mechanistic network, while subtyping of HCC tumors and characterization of their immune landscape are progressing towards becoming a standard clinical approach (Pinter et al., 2023). *In vivo* models –the cornerstone for modeling MASH-induced HCC– should be standardized to facilitate the accumulation of impactful findings on the role of T cell immunity during this malignancy. Nevertheless, to optimize the translational impact of basic *in vivo* research for this type of liver cancer, the scientific community should avoid piling on scarce findings arising from cancerous phenotypes that are relevant but do not accurately resemble the human MASH-induced HCC condition. Prioritizing the T cell subtypes to be further modeled in animals should also take precedence, which could be enabled by combining the characterization of the immune landscape of *in vivo* models through multi-omics technologies with patient derived data and clinical trial inclusive strategies. Future research could involve comparative Genome-Wide Association Studies (GWAS) to identify the in-between variations of the pathology among MASH-induced HCC patients and compose specific biological signatures for detecting and treating MASH-induced cancer. In summary, to uncover the precise functions of adaptive immunity in MASH and the transition to MASH-induced HCC, it is crucial to identify potential variations in the MASH-induced HCC phenotypes between murine models and humans. A recommended strategy involves adopting a combinatorial approach that leverages animal and human models to develop effective, patient-oriented therapeutics.
